# Host Plant Record for the Fruit Flies, *Anastrepha fumipennis* and *A. nascimentoi* (Diptera, Tephritidae)

**DOI:** 10.1673/031.008.4501

**Published:** 2008-06-02

**Authors:** Keiko Uramoto, David S. Martins, Rita C. A. Lima, Roberto A. Zucchi

**Affiliations:** ^1^ Instituto de Biociências, Universidade de São Paulo, Rua do Matão 277, Cep 05508-090, São Paulo, SP, Brazil; ^2^ INCAPER, Rua Afonso Sarlo, 160, Cep 29052-010, Vitória, ES, Brazil; ^3^ Escola Superior de Agricultura Luiz de Queiroz, Universidade de São Paulo, Av Pádua Dias, 11, Cep 13418-900, Piracicaba, SP, Brazil

**Keywords:** natural reserve, native host plants, *Cathedra bahiensis*

## Abstract

The first host plant record for *Anastrepha fumipennis* Lima (Diptera: Tephritidae) in *Geissospermum laeve* (Vell.) Baill (Apocynaceae) and for *A. nascimentoi* Zucchi found in *Cathedra bahiensis* Sleumer (Olacaceae) was determined in a host plant survey of fruit flies undertaken at the “Reserva Natural da Companhia Vale do Rio Doce”. This reserve is located in an Atlantic Rain Forest remnant area, in Linhares county, state of Espírito Santo, Brazil. The phylogenetic relationships of *Anastrepha* species and their hosts are discussed. The occurrence of these fruit fly species in relation to the distribution range of their host plants is also discussed.

## Introduction

The hosts of more than 50% of all *Anastrepha* species are unknown, many of them are probably native species (Norrbom et al. 1988). Of the 213 *Anastrepha* species known, 47% occur in Brazil, although there are host-records for less than half of these species (Zucchi 2007). *Anastrepha fumipennis*Lima, 1934 (Diptera: Tephritidae) and *A. nascimentoi*Zucchi, 1979 are known exclusively from Brazil. *A. fumipennis* was only known from two type-specimens collected in Rio de Janeiro state, before being rediscovered in a fruit fly survey undertaken in commercial papaya orchards in the state of Espírito Santo (Martins et al. 2000). *A. nascimentoi* was recorded in the states of Bahia (Zucchi 1979; Nascimentoi et al. 1981) and Espírito Santo (Martins et al. 2005). However, the host plant was unknown for both fruit fly species.

To survey native fruit fly hosts an assay was undertaken in the “Reserva Natural da Companhia Vale do Rio Doce”, an Atlantic Rain Forest remnant area in Linhares county, Espírito Santo state, Brazil.

## Materials and Methods

**Figure 1.**
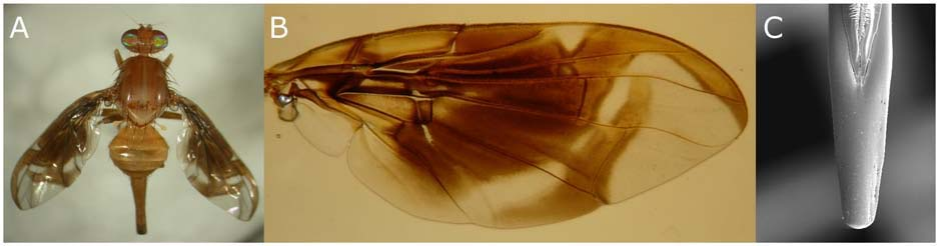
*Anastrepha fumipennis:* female (a), wing (b), aculeus tip (c).

Fruits, which had recently fallen or were picked from the tree, were sampled at the “Reserva Natural da Companhia Vale do Rio Doce” (19°06′ and 19°18′S; 39°45′ and 40°19′W). Vegetation in the Reserva corresponds to a secondary dense ombrophilous forest (Souza et al. 2002), situated on the surface of Tertiary mesas in the Barreira formation (lowland forest “mata de tabuleiro”), which is characterized by a sequence of low-elevation hills (28–65 m) and flat-bottom valleys (Vicens et al. 1998). Fruits of *Geissospermum laeve* (Vell.) Baill and *Cathedra bahiensis* Sleumer were collected, according to the fruiting season of each species, from February/2003 to July/2007. Fruits of both species were counted, weighed and placed in vials with vermiculite to obtain puparia, which were transferred to adult emergence cages. As *A. fumipennis* shows remarkable external morphological characters, females and males could be identified (Figures 1a, 1b, 1c).

**Figure 2.**
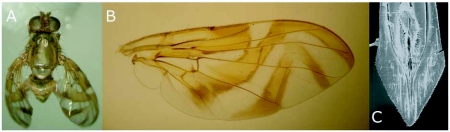
*Anastrepha nascimentoi:* female (a), wing (b), aculeus tip (c).

Normally, *A. nascimentoi* cannot be identified based on the male, however, as only females of this species were reared from the sample, the males of this sample were considered as also belonging to *A. nascimentoi* (Figures 2a, 2b, 2c). Voucher specimens were deposited in the collection of the Escola Superior de Agricultura Luiz de Queiroz, section of Entomology, Piracicaba, São Paulo. Plants were deposited in the Reserva herbarium.

## Results and Discussion

**Figure 3.**
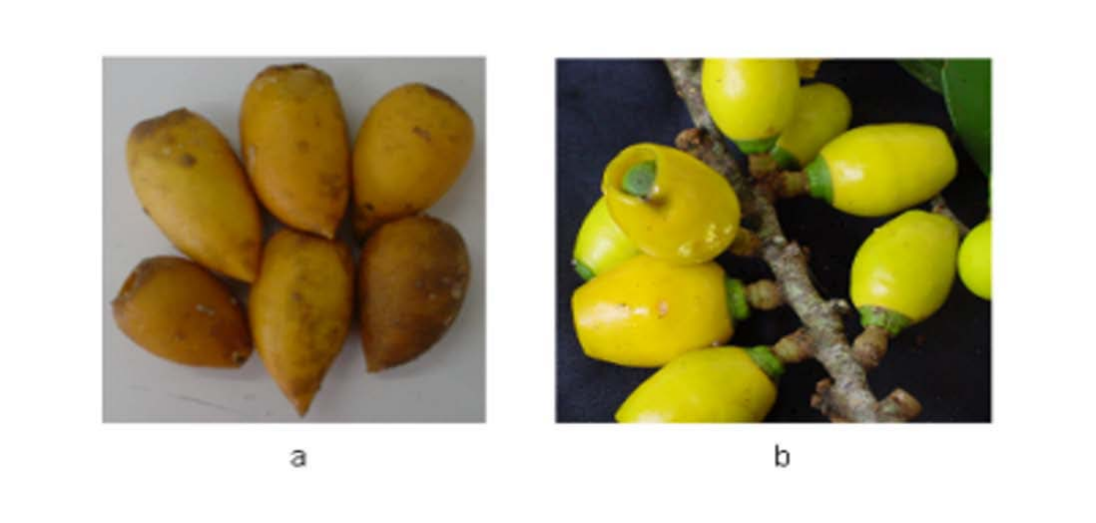
*Geissospermum laeve ripe fruits* (a), *Cathedra bahiensis* fruits (b).

248 fruit tree species belonging to 51 families were sampled totalizing 330 fruit samples (Uramoto 2007). Among these, three samples of *Geissospermum laeve* (Vell.) Baill. (Apocynaceae), known as “pau-pereira”, were collected in March 2003, February 2004 and 2006, in addition to one of *Cathedra bahiensis* Sleumer (Olacaceae), known as “baleira”, collected in January 2004. *G. laeve* is of common occurrence in the reserve, fruiting from December to March. The peel of the ripe fruit is brownish and the pulp very soft (Figure 3a). *C. bahiensis* fruits are small, with a yellow peel (Figure 3b). The fruiting period is from December to January.

Of three samples of *G. laeve,* adults of *A. fumipennis* (26 ♀ and 27 ♂) emerged from only one. The thin peel and soft pulp of *G. laeve* fruits are appropriate for *A. fumipennis* egg-laying, since the aculeus of this species has a rounded truncate tip, without serrations (Figure 1c). Specimens of *A. nascimentoi* (2 ♀ and 3 ♂) were reared from a single sample of *C. bahiensis* fruits. According to the host plant database (Norrbom 2004), no host was known for these two *Anastrepha* species so far. Therefore, this is the first host-plant record for both species. *A. fumipennis* is placed in the *grandis* species group as it shares apomorphies with *A. atrigona* Hendel, one of the species of this group (Norrbom et al. 1999). Recently, *A. atrigona* was also obtained from another species, *G. argenteum* Woodson (Apocynaceae), collected in the state of Amapá (Xavier et al. 2006). Although *A. nascimentoi* is not sorted in any species group, it may belong to the *spatulata* group, which is mainly associated with Euphorbiaceae and Olacaceae (Norrbom et al. 1999). Thus, these findings reinforce the hypothesis that host-plant associations of *Anastrepha* appear to be correlated with phylogenetic relationships within the genus (Norrbom et al. 1999). The mean fruit weight and infestation index values were 41.34 g and 0.32 puparia.g-1 for *G. laeve* and 13.40 g and 0.06 puparia.g-1 for *C. bahiensis.*

It is most likely that the host for these *Anastrepha* species was unknown because they maintain a specific association with their native hosts. This would also explain why their hosts were discovered only when native fruits were surveyed in a natural reserve. Considering *G. laeve* distribution, it is likely that *A. fumipennis* also occurs in the states of Amazonas, Bahia and the Federal District (state of Goiás), in addition to Rio de Janeiro (Lima 1934) and Espírito Santo (Martins et al. 2000). Likewise, *A. nascimentoi* may also occur in the state of Pernambuco, besides Bahia (Zucchi 1979; Nascimento et al. 1981) and Espírito Santo (Martins et al. 2005), as mentioned above coincides with the distribution of its host plant (New York Botanical Garden 2007). Recently *A. nascimentoi* was also recorded in the state of Rio de Janeiro, in Araruama (Aguiar-Menezes et al. 2006). This municipality is close to the border of the state of Espírito Santo, where *C. bahiensis* occurs.
